# Evidence-based Comprehensive Approach to Forearm Arterial Laceration

**DOI:** 10.5811/westjem.2015.10.28327

**Published:** 2015-12-11

**Authors:** Janice N. Thai, Jose A. Pacheco, David S. Margolis, Tianyi Swartz, Brandon Z. Massey, John A. Guisto, Jordan L. Smith, Joseph E. Sheppard

**Affiliations:** * University of Arizona Medical Center, Department of Surgery, Division of Vascular Surgery, Tucson, Arizona; † National Autonomous University of Honduras School of Medicine, Tegucigalpa, Honduras; ‡ University of Arizona Medical Center, Department of Orthopaedic Surgery, Tucson, Arizona; § University of Arizona Medical Center, Department of Surgery, Tucson, Arizona; ¶ The Institute for Plastic Surgery, Tucson, Arizona; || University of Arizona Medical Center, Department of Emergency Medicine, Tucson, Arizona

## Abstract

**Introduction:**

Penetrating injury to the forearm may cause an isolated radial or ulnar artery injury, or a complex injury involving other structures including veins, tendons and nerves. The management of forearm laceration with arterial injury involves both operative and nonoperative strategies. An evolution in management has emerged especially at urban trauma centers, where the multidisciplinary resource of trauma and hand subspecialties may invoke controversy pertaining to the optimal management of such injuries. The objective of this review was to provide an evidence-based, systematic, operative and nonoperative approach to the management of isolated and complex forearm lacerations. A comprehensive search of MedLine, Cochrane Library, Embase and the National Guideline Clearinghouse did not yield evidence-based management guidelines for forearm arterial laceration injury. No professional or societal consensus guidelines or best practice guidelines exist to our knowledge.

**Discussion:**

The optimal methods for achieving hemostasis are by a combination approach utilizing direct digital pressure, temporary tourniquet pressure, compressive dressings followed by wound closure. While surgical hemostasis may provide an expedited route for control of hemorrhage, this aggressive approach is often not needed (with a few exceptions) to achieve hemostasis for most forearm lacerations. Conservative methods mentioned above will attain the same result. Further, routine emergent or urgent operative exploration of forearm laceration injuries are not warranted and not cost-beneficial. It has been widely accepted with ample evidence in the literature that neither injury to forearm artery, nerve or tendon requires immediate surgical repair. Attention should be directed instead to control of bleeding, and perform a complete physical examination of the hand to document the presence or absence of other associated injuries. Critical ischemia will require expeditious surgical restoration of arterial perfusion. In a well-perfused hand, however, the presence of one intact artery is adequate to sustain viability without long-term functional disability, provided the palmar arch circulation is intact. Early consultation with a hand specialist should be pursued, and follow-up arrangement made for delayed primary repair in cases of complex injury.

**Conclusion:**

Management in accordance with well-established clinical principles will maximize treatment efficacy and functional outcome while minimizing the cost of medical care.

## INTRODUCTION

Upper extremity arterial injuries constitute up to 50% of peripheral vascular injuries.^1^–^2^ Penetrating injury to the forearm is a less common subset of upper extremity trauma. Lacerations of the forearm and wrist by knife, glass or machinery (often from occupational injury), are frequent to both the radial and ulnar arteries. Musculotendinous and nerve structures are also commonly injured (complex laceration). Emergency physicians and trauma surgeons constitute the front-line providers who direct initial management, where availability of hand subspecialty provides an additional level of expert care. The literature is replete with operative management strategies with hard signs, but sparse on conservative management without surgical intervention, a service often not available at rural emergency facilities. Conservative nonoperative management of lacerated forearm arterial injuries in accordance with well-established clinical principles will maximize treatment efficacy and functional outcome while minimizing healthcare costs.

A bleeding forearm involving arterial laceration presents dramatically and demands prompt attention. The presence of pulsatile bleeding is a hard sign of arterial injury. The first step in the management of this condition is to provide active hemorrhage control. Knowledge of vascular anatomy as well as the mechanisms of bleeding and coagulation will aid the treating physician in the management of arterial bleeding. The purpose of this review is to provide a thorough and systematic approach to management of forearm arterial laceration without emergent or urgent operative intervention. Inherent is the need for good communication and understanding between the treating emergency or trauma physician and the hand specialist consultant.

## DISCUSSION

### Arterial Anatomy

**Figure 1:**
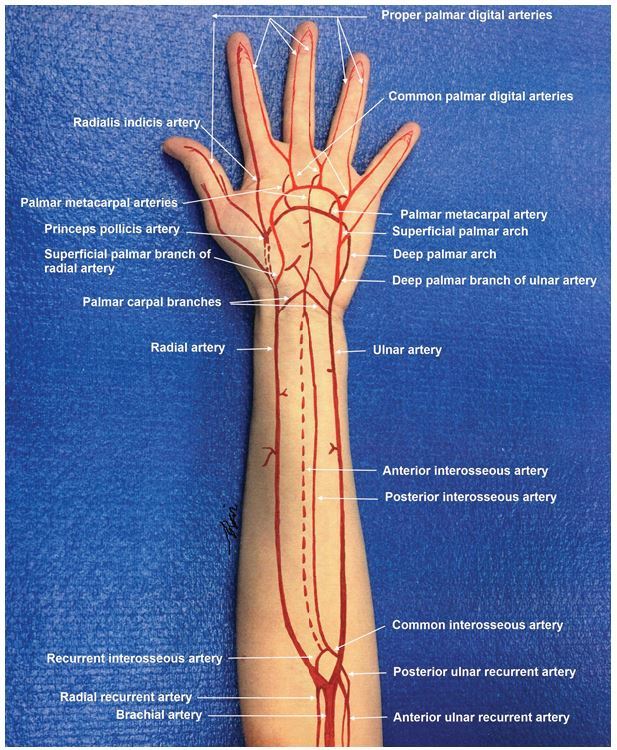
Arterial circulation in the forearm and hand.

In the proximal forearm, the brachial artery bifurcates at the level of the radial tuberosity into the radial and ulnar arteries. These arteries have recurrent branches that anastomose with the upstream brachial artery branches to form a network of rich collaterals around the elbow. The ulnar artery gives off the common interosseous artery, which immediately gives rise to the anterior (volar) and posterior (dorsal) interosseous branches that run on either side of the interosseous membrane. In the forearm the radial artery lies in proximity and medial to the superficial branch of the radial nerve, and the ulnar artery is joined in its course by the ulnar nerve. At the wrist, the radial artery traverses the anatomical snuffbox, gives off a superficial palmar branch that contributes to the superficial palmar arch, then winds dorsally around the wrist across the carpal bones (scaphoid and trapezium). In the hand, the radial artery travels dorsally through the first interosseous webspace and across the palm deep to the adductor pollicis muscle to join the deep palmar arch. The ulnar artery courses superficial to the transverse carpal ligament and deep to the palmar carpal ligament at the wrist, gives off a deep palmar branch and then continues on superficial to the flexor tendons to form the superficial palmar arch, the dominant arterial arcade supplying the hand. The hand has a robust collateral network comprised of the deep and superficial palmar arches, which derive their main contributions from the radial and ulnar arteries respectively (Figure 1). Approximately 20% of the population has an incomplete or interrupted superficial or more rarely deep palmar arch, with an absence of communication or anastomosis between the arterial branches of the arch. However, in the majority the superficial and deep palmar arches interconnect with each other and with the radial and ulnar circulations.^3^ The superficial palmar arch gives rise to the common palmar digital arteries, which in turn branch into the proper digital arteries to supply the fingers.

### Initial Patient Assessment

In a multi-trauma patient or a trauma patient in extremis, priority is given to initial resuscitation, stabilization, and identifying life-threatening injuries, according to the Advanced Trauma Life Support (ATLS) guidelines.^4^ Arterial hemorrhage may constitute such an injury. Further assessment must also be made of the viability and perfusion of the distal limb as described below.

## METHODS OF HEMORRHAGE CONTROL

### Manual Direct Digital Compression

Of the various methods for achieving hemostasis in a briskly bleeding wound, manual direct digital pressure over the bleeding artery is safe and effective.^5^ However, this method requires investment of sufficient time to obtain hemostasis, ranging from five to 15 minutes or more of consistent application of pressure without interruption and is often successful.^6^ Disturbance of the wound by manipulation or additional inspection will destabilize forming clot and contribute to additional blood loss and time to hemostasis. Heavy dressings will be ineffective if the point of bleeding is not precisely compressed.

### Temporary Tourniquet Application and Wound Closure

The next optimal method of achieving hemostasis is by temporarily applying an antebrachial pneumatic tourniquet for distal forearm laceration or brachial tourniquet for proximal forearm laceration. The correct pressure is the minimum amount required to produce a bloodless field. In an adult, upper extremity pressure 30–70mmHg higher than the systolic pressure may be sufficient to suppress arterial flow. An appropriate tourniquet pressure is around 250mmHg in adults and between 100 to 200mmHg in children, and adjusted for patient size and systolic pressure.^7^ If a pneumatic tourniquet is not readily available, a standard blood pressure cuff can be used, with clamping of the cuff with hemostats to maintain pressure. Ideally, before the cuff is inflated the arm is exsanguinated by elevation and distal-to-proximal centrifugal wrapping with an Esmarch bandage or tight ACE wrap. Tight compressive dressings should be applied over the wound. When the bleeding is adequately controlled under tourniquet pressure, the lacerated wound should be promptly inspected, debrided of foreign material, irrigated with normal saline, and promptly closed with running non-absorbable monofilament suture under local anesthesia. After wound closure, a compact compressive gauze dressing should be applied and reinforced with elastic bandage wrap and the tourniquet released. This method effectively achieves hemostasis by way of tissue tamponade. Inflation time should be kept to a minimum. Accurate tourniquet pressure monitoring avoids excessive pressure to nerve structure underneath, which is the major source of tourniquet-related pain. Although, as a generally accepted safe limit, the normal forearm and hand can withstand a tourniquet time of up to two hours without ischemic sequelae,^8^,^9^ bleeding control solely by tourniquet is discouraged. Prolonged tourniquet use defeats the rich collateral network that maintains perfusion of an already compromised circulation to the distal forearm and hand.^9^ Moreover, when a tourniquet is used in an awake patient, pain may limit the duration of application and utility.

Arm elevation, for example by a cast stockinette sleeve rolled onto the arm with the other end tied to an elevated support such as an IV pole, may be used to stabilize the wound and decrease intravascular hydrostatic pressure, minimizing residual bleeding.

The close anatomic relationship between artery and nerve especially on the ulnar aspect of the wrist results in a high probability of nerve injury when attempts are made to place clamps or suture ligature on the bleeding artery. Blind application of clamps and ligatures in the bleeding wound risks iatrogenic injury to nerves including the superficial branch of the radial nerve, palmar cutaneous branch of the ulnar nerve and the ulnar nerve itself. Distally, intimacy between the common and digital arteries and nerves guarantees cross ligation. Therefore, clamps and suture ligature on bleeding points are not a recommended method of achieving acute hemostasis in this setting. Similarly, wound exploration should be performed cautiously to avoid iatrogenic injury to adjacent structures.^10^

Other causes of wound bleeding include injury to veins and tributaries, as well as tearing of small vessels between tissue planes. These bleeding sources can be easily controlled with the same above-mentioned principles and methods of achieving hemostasis, with the exception that venous ligation may be considered in safe anatomic locations.

### Comprehensive Physical Examination

After control of arterial hemorrhage, a thorough and systematic physical examination in the injured forearm and hand should be performed including comparison made to the uninjured hand, to evaluate perfusion and neurological status, as well as for concomitant bone, nerve or tendon injury. This assessment directs the subsequent treatment and management. The duration and pressure of a tourniquet, as well as use of local anesthetics must be documented to avoid compromise of subsequent examinations and inadvertent prolonged ischemia.

### Vascular Examination

A complete vascular examination involves assessing for any clinical evidence of ischemia or vascular insufficiency to the hand. This includes palpation of the radial and ulnar pulses. The radial artery, in its superficial location, is easily palpable at the wrist, while the ulnar artery may not be as easily detected. If pulses are not palpable, interrogation with a hand-held Doppler device should be performed seeking brisk, triphasic Doppler signals. It is important to compare the pulse or Doppler examination to the non-injured hand. An asymmetric pulse is almost always abnormal. Documentation of an intact radial or ulnar artery is important in a lacerated forearm.

**Figure 2:**
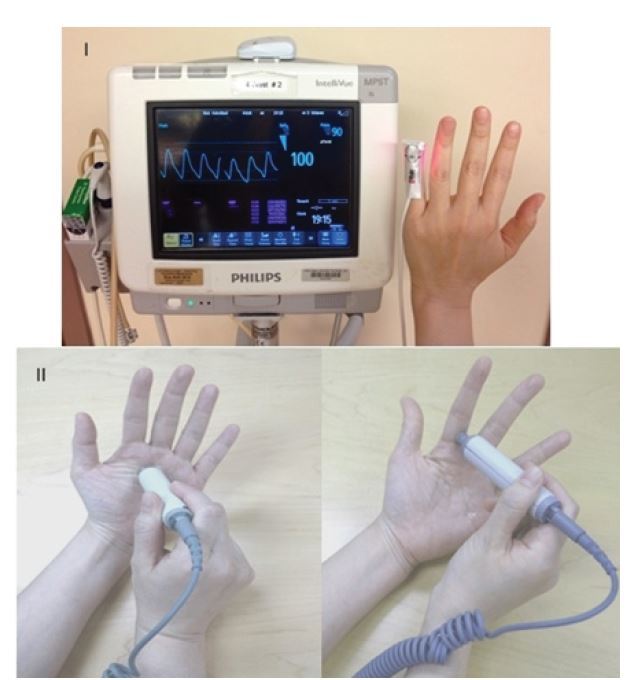
I: Digital pulse oximetry measurement; II: Doppler interrogation of palmar arch and digital artery.

An ischemic hand is painful and the symptom of pain should raise suspicion of vascular insufficiency. Motor and sensory deficits can also result from arterial insufficiency, ranging from paresthesia to weakness and paralysis. Use skin color, turgor, temperature, backflow and distal fingertip or nail bed capillary refill time to assess perfusion to the hand. Inspection and palpation of an ischemic hand will reveal paleness or pallor, bluish discoloration or cyanosis, skin mottling and coolness as compared to the rest of the body. A delayed or asymmetric finger capillary refill time >2 seconds signifies decreased perfusion. Assessment of tissue oxygen saturation with pulse oximetry, using a pulse oximeter sensor is an important additional perfusion assessment method. It is quick and reliable, and should be measured in more than one digit (Figure 2). Asymmetry or values significantly less than alternate sites of measurement would suggest hypoperfusion. Serial readings can be helpful for continuous assessment to detect any deterioration from the baseline assessment. Finger pulp skin temperature can be measured with a temperature probe. A reading <30°C (<86°F) may indicate decreased perfusion. It is reliable, widely available and simple.

### Adjunct Vascular Examination

While not a priority exam, the Allen’s test, originally described in 1929, can be performed to evaluate the integrity of the palmar arch and palmar collateral circulation.^11^ A variation of this technique exists and is known as the modified Allen’s test.^12^,^13^ To perform the Allen’s test, the examiner will first occlude the radial artery with one hand, followed by occluding the ulnar artery with the other hand. The patient’s hand is then clasped in a tight fist to exsanguinate the palmar arch. The hand is then relaxed. To assess the integrity of the palmar arch circulation, either one of the radial or ulnar artery compression is released while the other remains occluded. If the uncompressed artery and palmar arch is intact, the hand will blush with return of circulation. An Allen’s test is said to be positive when the blush occurs, demonstrating patency of the artery tested and integrity of the palmar arch circulation. A delay in filling time >6 seconds is widely used as a cut-off to imply an incomplete or interrupted arch. However, this is not an absolute discriminatory test as controversy exists surrounding its reliability in predicting hand ischemia.^14^

Alternatively, Doppler signal interrogation of the palmar arch and digital arteries can be performed with a hand-held Doppler (Figure 2). The Doppler Allen’s test can also be conducted to evaluate continuity of the palmar arch.^15^,^16^ In theory, an intact palmar arch circulation is required for maintaining adequate vascular perfusion to the hand in the setting of an injury to one of the two forearm arteries. Common sense would dictate that repair of an injured artery is mandatory with an incomplete palmar arch. The presence of intact palmar arch communication, however, is seldom documented in a traumatic setting. Therefore, the presence of a warm and well-perfused hand without evidence of acute ischemia on physical examination is often used in-lieu of an objective finding to imply an intact arch.

### Neurological Examination

A complete neurological examination of the hand should assess the integrity of the major peripheral nerves, both sensory and motor function. This includes the radial nerve (branch posterior interosseous nerve), median nerve (branch anterior interosseous nerve) and ulnar nerve. A thorough, systematic approach should be routine on every injured forearm and hand, and can be done rapidly in an awake and cooperative patient.

### Motor Examination of the Hand

**Figure 3:**
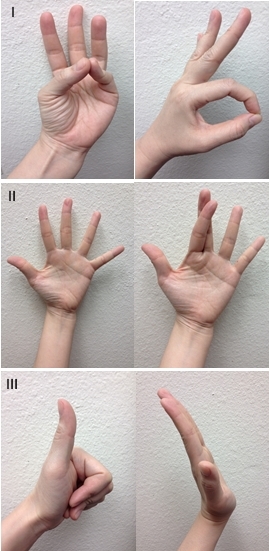
Motor examination of the hand. I: Median nerve. II: Ulnar nerve. III: Radial nerve.

Focused motor examination includes evaluation of extrinsic forearm and intrinsic hand muscle innervation. Evaluation of median nerve integrity includes thumb opposition with little finger, and flexion of thumb interphalangeal (IP) joint with index finger proximal IP (PIP) joint to form an “OK” sign. Ulnar nerve evaluation includes abduction of fingers by spreading them apart, and criss-crossing the index and third finger. Radial nerve evaluation includes thumb extension to make a “thumbs-up” sign, dorsal wrist extension and extension of fingers at the metacarpophalangeal (MCP) joint (Figure 3). The radial nerve innervates the forearm muscles that provide extension of the wrist, thumb, and all finger MCP joints. The median nerve has dual intrinsic and extrinsic innervation. In the hand, the motor branch of the median nerve provides innervation for thumb palmar abduction. In the forearm, it innervates the muscles that flex the thumb, wrist, and all the PIP and DIP joints of the index and middle fingers. The ulnar nerve also has dual innervation. It provides the dominant innervation to the intrinsic muscles of the hand, flexion of the MCP joints and extension of the IP joints of the fingers and adduction of the thumb. In the forearm, it innervates the muscles that flex the wrist and DIP joints of the ring and little fingers.

### Sensory Examination of the Hand

**Figure 4:**
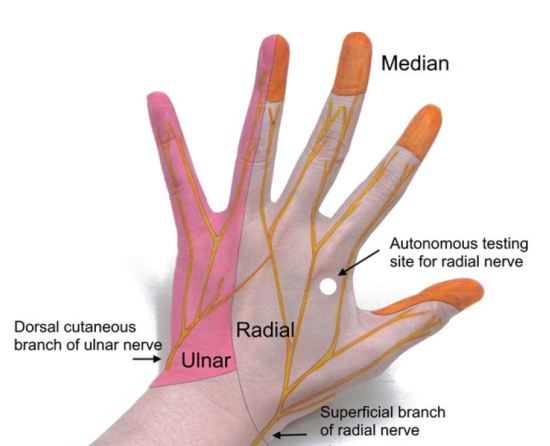
Cutaneous innervation of the hand.

**Figure 5:**
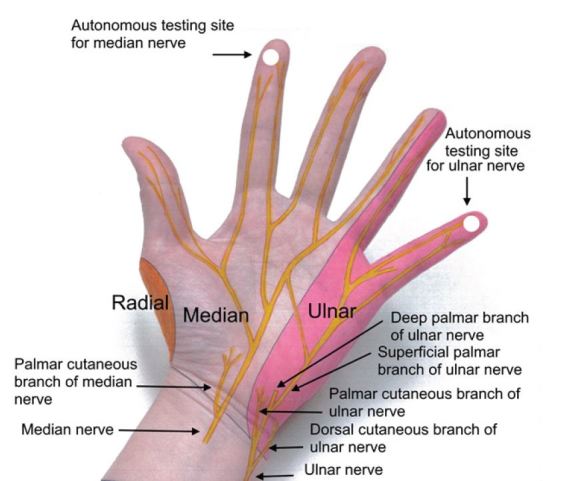
Cutaneous innervation of the hand.

Focused sensory examination includes assessment of sensation to light touch and pin-prick as well as two-point discrimination test, with ≤5mm as normal.^17^ The median nerve sensory distribution includes the volar aspect of hand from the thumb to the radial half of the ring finger; and the dorsal aspect of index, middle, and radial half of the ring finger from the PIP joint to the tip of the finger. The ulnar nerve sensory distribution includes the dorsal and volar sides of the ulnar aspect of the hand and medial half of the ring finger and the entire little finger. The radial nerve sensory distribution includes the dorsal aspect of the radial two-thirds of the hand and thumb; and the dorsal aspect of the thumb, index, middle, and radial half of the ring finger to the PIP joint (Figures 4 and 5). Sensations of numbness or tingling should also be elicited to disclose subtle subjective signs of nerve injury or early compartment syndrome.

### Musculoskeletal Examination

Wrist joint and skeletal stability through palpation and gentle manipulation, as well as active and passive range of motion, should be assessed to evaluate for concomitant bone, muscle, tendon or ligament injury. Tendon integrity and muscle function examination includes extensors of the wrist, thumb and digits on the dorsal aspect, and similarly flexor mechanisms on the volar aspect. In addition, ulnar and radial deviation of the wrist should be assessed. Knowledge of local anatomy will permit a limited direct assessment of the identity and integrity of local structures. Documentation of nervous, myotendinous or ligamentous injury is important, and will be helpful for completion of possible later reconstructive procedures. Focused physical exam and identification of associated dysfunction will corroborate anatomic findings. Clinically appropriate suspicion of compartment syndrome should exist during examination and is discussed further below.

## OTHER CONSIDERATIONS

### Operative Repair or Ligation?

Operative management is required in the case of persistent bleeding after exhaustion of conservative methods of hemorrhage control. This may occur where hemodynamic instability persists despite adequate resuscitation. The decision to perform a surgical ligation or repair of the artery is at the surgeon’s discretion. It is well-established in the literature that nerve injury determines the long-term functional disability of the hand, not arterial injury, which may be associated with non-debilitating exercise or work-induced hand claudication, cold intolerance, or presence of other disabling ischemic symptoms.^18^–^26^ Isolated laceration of either the radial or ulnar artery is usually not critical, given the rich collateral anastomoses found in the hand. It is safe and acceptable in a well-perfused hand to ligate, if necessary, a distal forearm artery as long as there is one remaining patent artery and the palmar arch circulation is intact. Attempts at vessel repair have been documented at 50–77% patency rate.^27^,^28^ Ligation, however, is only safe in the operating room with adequate exposure and control. It is not to be attempted in an emergent or urgent setting in an uncontrolled environment, as to avoid iatrogenic nerve injury. Rather, hemostasis should be attempted with utilization of the proper techniques described above. Arterial repair is mandated if both the radial and ulnar arteries are injured, or if suspicion of an incomplete arch exists.

### Complex Laceration Injury

A large body of evidence-based literature exists to affirm the safe clinical practice of delayed primary repair involving traumatic injuries to nerves and tendons of the forearm and wrists.^29^–^31^ Priority should be centered on control of hemorrhage. Delayed primary repair can be performed at an outpatient setting for any associated injuries by a hand specialist consultant, when proper staff equipment and operative time may be more effectively available.

### Diagnostic Imaging Studies

Imaging work-up is dictated by clinical assessment of history, physical exam and/or mechanism of injury. Plain radiographs will assist in identification of associated fracture or foreign body. Advanced imaging techniques such as computed tomography angiography or magnetic resonance angiography are not indicated.^32^,^33^ Invasive contrast arteriography is unnecessary.^34^

### Acute Compartment Syndrome

Although a rare occurrence in an isolated arterial laceration injury, acute compartment syndrome in the forearm may occur, especially in the setting of associated injuries involving fractures, extensive soft tissue injury or crush injury.^35^ The forearm comprises three separate fascial compartments: volar, dorsal, and the lateral mobile wad. The hand has 10 fascial compartments: four dorsal and three volar compartments, an adductor pollicis compartment as well as the thenar and hypothenar compartments. The diagnosis of acute compartment syndrome is a clinical one. Cardinal signs of compartment syndrome include swollen and taut muscle compartment(s), pain out of proportion to injury or severe pain on passive muscle stretch with digital extension. Neurological deficit is an important clinical feature with paresthesias indicating early nerve ischemia, and paresis or paralysis being late features of nerve and muscular dysfunction. The clinical diagnosis may be substantiated with intracompartmental pressure measurements. A finding of measured intracompartmental absolute pressure at ≥30mmHg, or delta-pressure (muscle perfusion pressure) with a differential between systemic diastolic pressure and compartment pressure ≤20mmHg, is an indication for fasciotomy in patients with a supporting physical examination or who are unable to be adequately examined for any reason.^36^ Emergent forearm fasciotomy is indicated for decompression of fascial compartments to prevent irreversible muscle and nerve damage. Surgical techniques for volar compartment decompression include the ulnar approach, or the radial (Henry) approach and its modified version. The dorsal compartment and mobile wad can be decompressed with a single midline dorsal longitudinal incision. A carpal tunnel release should be considered at the time of fasciotomy.^37^–^40^

### Relevant Medical Conditions

Patients with advanced hepatic disease, or taking anticoagulants such as warfarin may have an elevated international normalized ratio (INR) and will exhibit impaired hemostasis. These patients should have their INR normalized. However, in rare situations involving patients on warfarin therapy, the risk benefit ratio of decreasing the INR should first be considered. Such patients include those with a history of vascular graft thrombosis or those with a mechanical heart valve. This decision should be made on an individual basis with sound clinical judgment. Discussion with appropriate specialists may be helpful.

Patients with hypotension due to volume depletion should first be adequately resuscitated with isotonic fluid. Findings of low hemoglobin or hematocrit level may be treated with blood transfusion. Patients presenting with hypertension should be treated with anti-hypertensive medications to normalize their blood pressure in order to facilitate hemostasis.^41^

### Tetanus Prophylaxis and Prophylactic Antibiotics

Tetanus prophylaxis should be considered. A booster dose of the tetanus toxoid is given to previously immunized individuals, and tetanus toxoid plus tetanus antitoxin containing human tetanus immune globulin to non-immunized individuals.

Broad spectrum antibiotics may be considered in extensive or contaminated wounds. One intravenous dose, followed by several days of oral administration may reduce the risk of wound infection. Clean or minimally contaminated lacerated wounds may only require orally administered antibiotics. Systemic antibiotics effective against skin flora such as a first-generation cephalosporin is typically used (e.g. Cephalexin 500mg p.o. QID).^42^

### Expert Consultation and Hand Immobilization

Consultation with a hand specialist should be obtained immediately following determination of the extent of injury to discuss further management. Application of a forearm and hand splint is recommended for those with concomitant tendon or bone injury to reduce swelling, provide stabilization and relative comfort, and allow early mobilization of uninvolved joints. The wrist may be splinted in 0° to 30° of extension (dorsiflexion).^43^ Outpatient follow up with a hand specialist should be arranged for dressing change, wound inspection and suture removal in the setting of an isolated arterial injury or complex injury for delayed primary repair.

## CONCLUSION

An optimal management of forearm arterial laceration is based on basic principles of achieving hemostasis. Operative treatment algorithms are widely practiced and remain the current trend in management at urban trauma centers. However, the need for emergent or urgent surgical intervention is not justified based on the available evidence and from a cost-effectiveness standpoint. Furthermore, trauma and hand surgery specialists are not widely available in the rural settings. Emergency clinicians can be confident in the evidence-based nonoperative approach when faced with an arterial injury involving a forearm laceration. This includes isolated or complex laceration with concomitant nerve and/or tendon injury. Attention must first be given to the presence of other life-threatening injury, and to the initial resuscitation and hemodynamic stabilization of the patient. Concomitantly, measures are taken to stop additional blood loss. This can be accomplished most effectively by a combination of direct manual pressure, temporary pneumatic tourniquet inflation for proximal control, followed by prompt wound debridement and rapid wound closure. The sole or prolonged use of tourniquet is discouraged, as is blind clamping or attempted ligation of an artery. A complete and thorough assessment of the hand should include neurovascular status and musculoskeletal integrity. Documented critical ischemia must be addressed expeditiously with surgical restoration of arterial perfusion. In a well perfused and non-ischemic hand, the presence of one intact artery is adequate to sustain viability. Long-term functional disability will not be compromised, provided the palmar arch circulation is assessed to be intact. Concomitant injuries to nerves and tendons in a complex laceration can be safely repaired in a delayed fashion. Immediate surgical exploration is not mandatory if bleeding can be stopped with conservative compressive maneuvers and a complete physical examination of the hand is performed. The appropriateness and safety of an outpatient strategy is validated with evidence-based literature. The evolution of an acute compartment syndrome must be monitored in the presence of suggestive clinical signs. A hand specialist consultation should be obtained early after adequate control of bleeding to guide subsequent therapeutic requirements. Adherence to these basic principles of management will not only streamline patient management but will also decrease cost and optimize clinical outcome.
